# Evaluation of outcomes, costs, and feasibility of home-based geriatric rehabilitation after inpatient rehabilitation: study protocol of the “Better@Home” multicentre prospective cohort study with historical control group

**DOI:** 10.1186/s12877-025-06573-6

**Published:** 2025-11-28

**Authors:** M.C. Pol, E.F van Dam van Isselt, A.J. Doornebosch, C.J. Gamble, M. Vaz, W.G. Groen, M.W.M. de Waal, S.M. Debeij, F. Innocenti, M. Hiligsmann, J.C.M. van Haastregt

**Affiliations:** 1 Department of Medicine for Older People, Amsterdam University Medical Center, Location Vrije Universiteit Amsterdam, Amsterdam, The Netherlands; 2 Amsterdam Public Health, Ageing & Later Life, Amsterdam, The Netherlands; 3 Research group Occupational Therapy: Technology and Participation, Faculty of Health, Sport and Physical Activity, Amsterdam University of Applied Sciences, Centre of Expertise Urban Vitality, Amsterdam, The Netherlands; 4 University Network of Organizations for Care for Older Adults, Amsterdam University Medical Center, Amsterdam, The Netherlands; 5 University Network for the Care sector South-Holland, Leiden University Medical Center, Leiden, the Netherlands; 6 Department of Public Health and Primary Care, Leiden University Medical Center, Leiden, the Netherlands; 7 Department of Health Services Research, Faculty of Health Medicine and Life Sciences, CAPHRI Care and Public Health Research Institute, Maastricht University, Maastricht, the Netherlands; 8 Limburg Living lab in Ageing and Long-term Care, Maastricht University, Maastricht, the Netherlands; 9 Amsterdam Movement Sciences, Ageing & Vitality, Rehabilitation & Development, Amsterdam, The Netherlands; 10 Department of Methodology and Statistics, Faculty of Health Medicine and Life Sciences, CAPHRI Care and Public Health Research Institute, Maastricht University, Maastricht, the Netherlands

**Keywords:** Geriatric rehabilitation, Home-based geriatric rehabilitation, Multicentre-prospective cohortstudy, Outcomes- costs-and feasibility

## Abstract

**Background:**

The increasing demands of an aging population, healthcare workforce shortages, financial constraints, and a shift in care perspectives call for rethinking geriatric rehabilitation (GR). To ensure GR remains sustainable, a transition towards home-based GR is proposed, reducing the need for prolonged inpatient GR. This study assesses the outcomes, costs and feasibility of the “Better@Home” program, in which home-based GR replaces part of inpatient GR.

**Methods:**

This multicenter cohort study is conducted in eight GR facilities in the Netherlands, implementing the Better@Home program. Core elements of this program include replacing part of inpatient GR with home-based GR, focusing on participation goals, using eHealth, promoting self-management, and fostering close collaboration among all care partners. Data is gathered through semi-structured interviews, questionnaires, group interviews, registration forms, and electronic patient files.

The Better@Home study is designed as a cohort study accompanied by a mixed-methods feasibility study. The study includes an outcome-and cost assessment. Within the cohort study, two evaluations can be distinguished. The first is a *comparative evaluation*, comparing the multicentre prospective Better@Home cohort with a historical control group on the primary outcome measure, as well as patient, family, and healthcare-related costs, from admission to completion of GR. The primary outcome measure of the *comparative evaluation* is independence in activities of daily living, assessed by the Barthel Index. The second is a *follow-up evaluation*, to assess the course of the outcomes and costs from GR admission to three months of follow-up after GR completion, solely in the Better@Home cohort. The primary outcome measure of the *follow-up evaluation* is participation, assessed by the Canadian Occupational Performance Measure. The *mixed-methods* feasibility study incorporates both quantitative and qualitative methods. It evaluates the program’s reach, performance according to plan, active engagement of patients and informal caregivers, barriers and facilitators affecting implementation, and the opinions of patients, informal caregivers, and professionals on the program.

**Discussion:**

This study offers insights into the potential of home-based GR. The multicentre and multilayered design enables a comprehensive evaluation of the Better@Home program’s outcomes, costs and feasibility, providing a basis for further optimization and upscaling of home-based GR.

**Supplementary Information:**

The online version contains supplementary material available at 10.1186/s12877-025-06573-6.

## Background

Due to population aging, shortages of healthcare professionals, pressures on healthcare budgets, and a shifting perspective on care, geriatric rehabilitation (GR) should be reconfigured [1–4]. GR is a multidimensional approach of diagnostic and therapeutic interventions to optimize functional capacity, promote activity, and preserve functional reserve and social participation in older people with disabling impairments [5].

To continue providing effective, affordable, and accessible GR for an increasing number of older people with a decreasing number of healthcare professionals, GR must be made future-proof. A crucial aspect of future-proof GR is providing ‘the right care in the right place’ through home-based rehabilitation after (shorter) inpatient rehabilitation, potentially with technological support [2]. First, this can contribute to a more efficient organization of care (e.g., reduced personnel and lower costs). Second, home-based GR is expected to positively affect older people because rehabilitation at home offers a realistic rehabilitation environment in which patients can practice their activities of daily living [6]. It is known that older adults often experience the transition from inpatient rehabilitation to home as very challenging [7]. After discharge, they truly realize the limitations they face in their homes. This manifests in a feeling of not being able to successfully continue their daily functioning and finding it challenging to organize meaningful participation in their home environment [7]. Home-based GR may help ease this transition because older adults can practice meaningful activities at home under the guidance of multidisciplinary GR professionals and with the assistance of (other) formal and informal caregivers. Sustainable strategies can be developed to resume life and remain at home longer despite experiencing limitations [8, 9].

Despite a broad consensus on the importance of home-based GR after (shorter) inpatient GR [5, 10], it is currently only used on a small scale in the Netherlands. There are various barriers, including regulatory and funding issues, and a lack of knowledge and skills to apply home-based GR in practice [11, 12]. There is insufficient understanding of how home-based GR after inpatient GR can be provided in a feasible and (cost-)effective way.

To overcome the barriers and challenges, we developed and implemented a home-based GR program in which home-based GR substitutes part of the inpatient GR. The program, called Better@Home, is based on current best practices in healthcare organizations in the Netherlands and results from previous research [13]. This includes knowledge on strengthening self-management skills and social networks through reablement [14, 15], the application of eHealth [16–18], and the development of care pathways [19, 20]. The core principles in the Better@Home program are: (1) replacing part of inpatient GR by home-based GR; (2) focusing on participation goals during both inpatient GR and home-based GR; (3) using eHealth to support rehabilitation; (4) promoting the patients’ self-management through the application of various reablement strategies; and (5) close collaboration between all care partners involved during the GR trajectory.

The aim of our study is to assess the outcomes, costs, and feasibility of home-based GR after shorter inpatient rehabilitation.

## Methods

### Study design

**Fig. 1:**
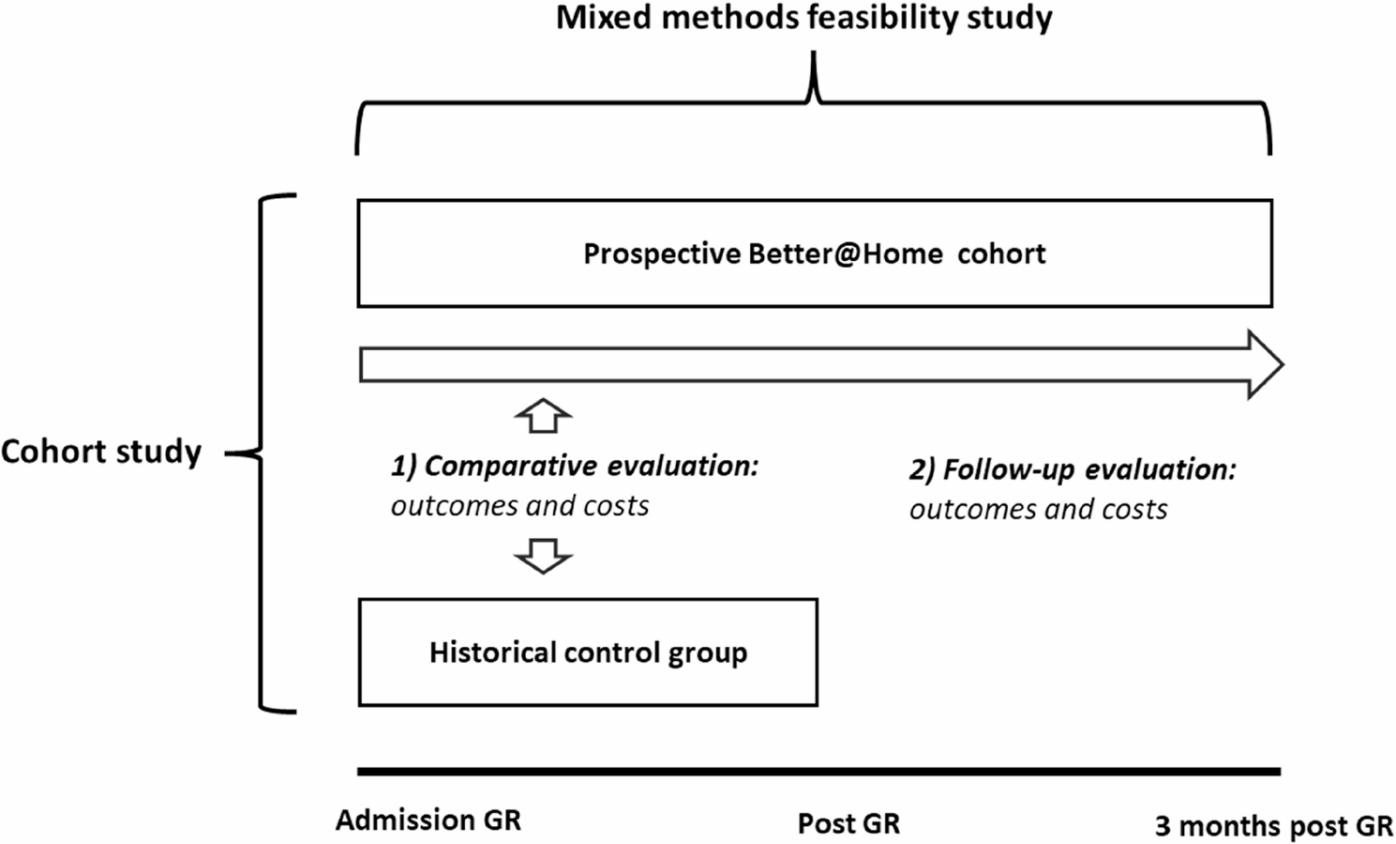
Schematic overview of study designs and cohorts in the Better@Home study

The Better@Home study is a cohort study accompanied by a mixed-methods feasibility study (Fig. 1). The Better@Home study includes an outcome assessment to assess the program’s outcomes and a cost assessment to assess the costs in relation to the outcomes. Within the cohort study, two evaluations can be distinguished: [1] a *comparative evaluation*, to assess the outcomes and costs from admission to completion of GR in the prospective Better@Home cohort receiving the Better@Home program, compared to a historical control group who received usual care; [2] a *follow-up evaluation*, to assess the course of the outcomes and costs solely within the prospective Better@Home cohort, from admission to three months post-GR. The *mixed-methods study* is to assess the feasibility of the Better@Home program, from admission to three months post-GR. The mixed-methods study incorporates both quantitative and qualitative methods. It evaluates the program’s reach, performance according to plan, active engagement of patients and informal caregivers, barriers and facilitators affecting implementation, and the opinions of patients, informal caregivers, and professionals on the program.

The current study was assessed by the Medical Ethics Committee of University Hospital Maastricht/Maastricht University (reference number 2023–3744) (see Ethics declarations). Written informed consent was obtained from the participants in the Better@Home cohort (patients, informal caregivers, and care professionals who participated in the questionnaire and/or interview). All patients’ data in the Care as usual cohort is received from the GR facilities anonymized, so it cannot be linked to specific persons by the research team.

### Setting and population

The Better@Home study is performed in eight GR facilities situated in the Netherlands, embedded in three university networks for the care sector: Limburg Living lab in Ageing and Long-term Care (AWO-L) on behalf of Maastricht University; University Network of Organizations for Care for Older Adults (UNO Amsterdam) on behalf of Amsterdam University Medical Center (location VUmc); and University network for the Care Sector South-Holland (UNC-ZH) on behalf of University Leiden, in which these organizations participate. GR facilities have been invited to participate if they were willing to develop and implement the Better@Home program in their GR facility. This could be organizations with and without prior experience in providing home-based GR.

The target population for the prospective Better@Home cohort consists of patients admitted to one of the eight participating GR facilities that implemented the Better@Home program. Patients are eligible for participation in the Better@Home cohort if they meet the following inclusion criteria: (1) admitted to one of the eight participating GR facilities between December 2023 and November 2024; (2) are considered eligible for the Better@Home program as assessed by the multidisciplinary team of each GR facility individually (based on their physical, cognitive and psychological functioning); (3) are living in the community; (4) are able to participate in an interview as assessed by the multidisciplinary team of each GR facility individually (based on their physical, cognitive and psychological functioning); (5) are able to speak and understand Dutch; and (6) are able to provide informed consent. Furthermore, the multidisciplinary teams of each GR facility individually assess the eligibility of each patient on a combination of secondary criteria based on local GR facility protocols: (1) each GR facility is free to choose sub-population(s) based on diagnosis to be eligible for the program. This can be a single diagnosis group, several diagnosis groups, or all patients admitted to GR; (2) practical considerations (such as travel distance from the GR facility to the patient’s home and home hygiene status); and (3) patient-related considerations (such as availability of professional home care and informal care, functional status and wishes of the patient). In addition, the primary informal caregiver of every patient included in the Better@Home program will be invited to participate in the study. A person is considered the primary informal caregiver if the patient indicates that they are the person primarily involved in the informal care and support for this patient. For the informal caregivers, no historical control group is available.

The matched historical control group represents patients who received usual care. This cohort consists of patients admitted to the participating GR facilities between January 2022 and June 2023—at least six months before the implementation of the Better@Home program—thereby minimizing contamination bias while ensuring a similar context of care. Furthermore, in order to maintain the integrity of the comparison, patients who received home-based GR prior to the implementation of Better@Home program will be excluded from the matched historical control group. Several participating GR facilities already offered home-based GR during the comparison period; including these patients could lead to contamination of the usual care group and an underestimation of the Better@Home program’s effects.

Matching of the historical control group will be performed as follows. Based on the scores for independence in activities of daily living, as measured by the Barthel Index (BI) at admission in the Better@Home cohort, BI strata will be constructed per diagnosis group in each facility. Subsequently, frequency matching will be conducted, whereby patients for the historical control group will be randomly selected per facility from each BI stratum within a diagnosis group. Data on diagnosis and BI at admission for the historical control group will be obtained from electronic patient files. Randomization of the historical control group will be conducted using an online randomization tool with two arms, a permuted block algorithm, and a fixed block size of 1. Differences between the Better@Home cohort and the historical control group will be accounted for by including relevant covariates in the analyses (see Data analysis). All patients’ data in the historical control group will be received anonymously from the GR facilities. At minimum, the same number of patients enrolling in the Better@Home program at each participating facility will be randomly included in historical control group. However, the target for the sample size of the historical control group is double that of the Better@Home cohort. This target is based on a sample size calculation for comparing two independent groups, assuming a two-tailed significance level of 5%, a power of 80%, an effect size varying between Cohen’s d = 0.51 (based on the ratio of a pre-determined aim of 120 included patients, 120:120) and Cohen’s d = 0.45 (based on the ratio 120:240), indicating a small-to-medium effect size, and includes a variance inflation factor of 2 to account for potential confounding factors.

Eligible patients for the Better@Home program will be invited individually by a multidisciplinary team member of each GR facility to participate in the study. The multidisciplinary team assesses eligibility within the first two weeks after admission to GR. After a patient indicates potential interest in participating in the study, a research team member contacts the patient to provide further detailed information on the study and check the inclusion criteria. Patients who agree to participate in the study are asked whether their primary informal caregiver can be approached for participation in the study. After obtaining the patient’s written consent, the informal caregiver is informed about the study and invited to participate.

### The Better@Home program

The Better@Home program is developed by the eight GR facilities participating in this study in collaboration with a learning network. The learning network includes project group members from the participating GR facilities (e.g., care professionals, managers, and policymakers), as well as (senior) researchers from the three university networks for the care sector. Moreover, representatives of national healthcare insurers, interest groups for older adult patients and informal caregivers, and other relevant stakeholders are also present in the learning network. The learning network facilitates the exchange of experiences and mutual learning across GR facilities.

A pilot project conducted by one of the eight participating GR facilities resulted in a draft version of the program, which led to the development of the final Better@Home program. The following five core components are present in the Better@Home program (Table 1): (1) Replacing part of inpatient GR by home-based GR; (2) Focusing on participation goals during GR; (3) Using eHealth to support rehabilitation; (4) Promoting patients’ self-management by applying Reablement strategies; and (5) Close collaboration between all relevant care partners involved in the GR trajectory. Each participating GR facility established a working group responsible for developing a site-specific, local Better@Home protocol containing the core components of the Better@Home program. While each of the five core components are mandatory, each GR facility retains the freedom to decide to what extent they implement each component in accordance with their local protocols. Each local Better@Home protocol is conducted by the multidisciplinary GR teams in each facility. In the Netherlands, these teams generally consist of physicians, nurses, physical-, occupational-, and speech therapists, dieticians, psychologists, and often complemented by social workers and nurse practitioners. The Better@Home program was gradually implemented between December 2023 and February 2024 and ended in November 2024.

**Table 1:** Core components of the Better@Home program

Core components Better@Home intervention program
*1) Replacing part of inpatient GR by home-based GR* • Each GR facility strives to reduce the duration of the inpatient GR with an average of 5 to 7 days and to replace this by a period of home-based GR. • The length and intensity of the home-based GR can vary and is tailored to the needs of the patient. • The home-based GR should at least partly consist of therapy provided at the patients’ home and can be supplemented with outpatient therapy at the GR facility and/or remote therapy using eHealth.
*2) Focusing on participation goals during both inpatient GR and home-based GR* • Using the Canadian Occupational Performance Measure (COPM), participation goals are set with the patient within the first two weeks after admission. • The participation goals should be relevant both in the inpatient and home-based phase of the GR trajectory and regularly evaluated until the end of the GR treatment. • The participation goals should be easy to understand and made visible to the patients and their informal caregiver(s).
3)*Using eHealth to support rehabilitation* • Exploring the available eHealth options, such as video calling (e.g., via a tablet equipped with software specifically designed for individuals with little to no digital experiences), rehabilitation apps for home exercises (e.g. Physitrack, and activity monitoring using a wearable sensor to monitor daily movement, all aimed at supporting remote rehabilitation. • Assessing the skills of the patient regarding the available eHealth options and providing support and/or training if needed. • Early introduction and involvement of the patient and the informal caregiver(s) to the eHealth application(s) during the inpatient phase. Aim for continued use of eHealth during the home-based phase of the GR trajectory.
*4) Promoting patients’ self-management by applying Reablement strategies* • Enhancing self-management skills and strategies. • Involving the informal caregiver(s) during the GR trajectory and providing support where necessary. • Assess the patients’ social network and support the patient to activate the network if desired and/or needed. • Providing advice and/or training on the use of aids, devices and home adjustments. • Assess the local or regional social services and organized (leisure) activities and how these could support the patient by participating in meaningful activities in daily life.
*5)Close collaboration between all care partners involved during the GR trajectory* • Determine the provisional start date of home-based GR within the first two weeks after admission (if possible). • Prepare the patient and informal caregiver(s) and discuss possible barriers or limitations for home-based GR. • Timely organization of the necessary home care with (structural) agreements with the relevant formal caregivers (f.e. home care organizations, general practitioners, allied health professionals and/or medical specialists in secondary care). • Timely preparation of transfer to follow-up care.

### Data collection

Details of the data collection procedure for the cohort- and mixed method feasibility study are presented below. Details are provided separately in accordance with the aim of the Better@Home study, which is to assess the outcomes, costs, and feasibility of home-based GR after shorter inpatient rehabilitation.

#### Outcome assessment

The outcome assessment entails a *comparative evaluation* comparing the Better@Home cohort with a matched historical control group at GR admission and immediately post-GR, as well as a *follow-up evaluation* assessing the course of all outcomes between GR admission and three months of follow-up, solely in the Better@Home cohort. Table 2 presents the background characteristics, outcomes measured at each time point and the data collection methods for the prospective cohort study.

**Table 2:** Background characteristics and outcome measures per time point

	Admission GR		Post GR		Follow-up 3 months
	**BH**	**CU**	**BH**	**CU**	**BH**
Patients					
*Outcome measures*					
Independence in daily functioning (BI)*	EPF	EPF	FI	EPF	FI
Performance in daily functioning (COPM-p)^#^	FI		FI		FI
Satisfaction with performance in daily functioning (COPM-s)	FI		FI		FI
Quality of life (EQ. 5D-5 L)	FI		FI		FI
B*ackground characteristics*					
Age	FI	EPF			
Sex	FI	EPF			
Educational level	FI				
Living arrangement	FI	EPF			
Diagnosis relevant for rehabilitation	RF	EPF			
Frailty (GFI)	FI				
Screening digital skills (Quick scan Pharos)	FI				
Informal caregivers					
*Outcome measure*					
Caregiver burden (CSI)	SQ		SQ		SQ
B*ackground characteristics*					
Age	SQ				
Sex	SQ				
Relationship to the patient	SQ				
Travel distance to the patient	SQ				

*BH* Better@Home cohort, *CU* Historical Control Group (care as usual), *EPF* Electronic Patient File, *FI *Face face-to-face interview patient, *RF* registration form care professionals, *SQ * structured questionnaire primary informal caregiver, *BI * Barthel-index, *COPM-p* Canadian Occupational Performance Measure-performance measure, *COPM-s * Canadian Occupational Performance Measure-satisfaction measure, *Eq. 5D* EuroQol health-related quality of life, *GFI* Groningen Frailty scale, *CSI* Caregiver Strain Index

*) Primary outcome measure comparative evaluation; ^#^) Primary outcome measure follow-up evaluation

#### Comparative evaluation

 The primary outcome measure for the *comparative evaluation* is independence in activities of daily living, assessed with the Barthel Index (BI) at admission to the GR facility and within two weeks post-GR, after completion of the GR trajectory. For both the Better@Home cohort and historical control group, the BI scores at admission are retrieved from the electronic patient files (EPF). The post-GR scores of the BI for the historical control group are also retrieved from the EPF. This data is routinely gathered by healthcare professionals of the participating GR facilities and stored in the EPF, most commonly in the Clinimetric Measurements tab. However, the post-GR scores of the BI for the Better@Home cohort are assessed by trained research assistants within 2 weeks of discharge. This is due to local GR protocols in which GR facilities do not routinely assess the BI after completion of home-based GR.

The BI, initially described by Mahoney and Barthel, is a 10-item measure of activities of daily living used in clinical practice to assess baseline abilities, quantify functional change after rehabilitation, and inform discharge planning. It comprises ten items: feeding, toilet use, bathing, grooming, dressing, bowel and bladder control, chair transfers, stair climbing, and ambulating [21]. In clinical practice, the patients are rated based on their medical records or direct observation. In a research setting, the patient is questioned during a face-to-face interview. Patients are rated on whether they can perform tasks independently, with assistance, or require assistance. The scoring on the modified version of the BI used in the present study ranges from 0 to 20. Higher scores indicate greater independence [21]. The BI’s structural validity, reliability, and interpretability are sufficient for measuring and interpreting changes in the physical function of geriatric rehabilitation patients [21–23].

In two of the participating facilities, the Utrecht Scale for Evaluation of Rehabilitation (USER) is used as an alternative for the BI [24, 25]. In these organizations, scores are converted from USER to BI [26]. This conversion is performed on a per-item level, as documented by DHU (De Hoogstraat Utrecht). For each BI-item, relevant scores on USER-items are matched, with the derived BI-item scores summed to the BI-total score. In the Netherlands, this conversion is common practice as GR facilities either use scores on the BI or the converted USER-scores to monitor progress in physical functioning.

#### Follow-up evaluation

 The primary outcome of the *follow-up* evaluation is performance in daily functioning, assessed with the “performance” subscale of the Canadian Occupational Performance Measure (COPM-p). A trained research assistant or care professional performs the COPM interview at admission to the GR facility, within two weeks post-GR after completion of the GR trajectory, and after three months of follow-up [27]. The COPM is a patient-centered, occupation-focused outcome measure that detects changes in perceived daily performance over time. The COPM consists of two subscales: the “performance” subscale (COPM-p) and the “satisfaction” subscale (COPM-s). Through a semi-structured interview, patients prioritize up to five daily activities they consider most important and would like to improve. The patients subsequently rate these activities on a 10-point scale regarding performance (COPM-p) and satisfaction (COPM-s) ranging from 1 to 10. Higher scores indicate a better performance or higher satisfaction. The mean scores for the subscales are obtained by dividing the ratings by the number of prioritized activities. Changes in scores are evaluated by asking the participant to rescore performance and satisfaction on the original prioritized activities. The COPM has excellent test-retest reliability and measures changes in daily activity performance [28–30]. In a systematic review of studies in GR, the COPM showed good content validity, good test-retest reliability, moderate interrater reliability, and moderate responsiveness [31]. A 1.3-point difference between pre- and post-measurement indicates a minimally clinically important difference [29, 31, 32].

We selected the COPM-p as the primary outcome for the *follow-up evaluation* as it fits with the Better@Home program, which focuses on participation in daily life. COPM-p scores are not routinely assessed by GR facilities in The Netherlands and could therefore not be used for the *comparative evaluation*, as this data was unavailable for the historical control group. The following four secondary outcomes are measured: independence in daily functioning (BI), patient satisfaction in performing daily activities (COPM-s) [27], and quality of life (EuroQol-5D-5 L) [33], which focuses on ‘health-related quality of life’ outcomes. The EuroQol-5D-5 L includes five dimensions: mobility, self-care, usual activities, pain or discomfort, and anxiety or depression. Each dimension has five levels, ranging from 1 to 5. A lower score indicates a better perceived quality of life. The fourth secondary outcome, the Caregiver Strain Index (CSI), is assessed among the primary informal caregivers of the patients in the Better@Home cohort [34, 35]. The CSI is a 13-item measure that assesses perceived care burden among informal caregivers. The scoring of the CSI ranges from 0 to 13. A higher score indicates a higher level of perceived strain related to care provision.

#### Cost assessment

The cost assessment entails a *cost-effectiveness analysis*, including data from both the Better@Home cohort and historical control group, and a *cost-analysis*, including data solely from the Better@Home cohort. The cost assessment is conducted from a societal perspective, which includes healthcare costs and patient and family costs. Due to the nature of the study participants (older adults, of whom the large majority are retired), productivity losses are not considered.

#### Comparative evaluation

 In the cost-effectiveness analysis, effects on the independence in daily functioning (BI) and healthcare-, patient & family costs are compared between the Better@Home cohort and historical control group at admission to GR and at post-GR, after completion of the GR-trajectory.

Healthcare costs are divided into costs made during inpatient GR and home-based GR. Healthcare costs during inpatient GR consist of the length of stay in the GR facility and (re)admissions to the hospital. This data can be accurately collected from the EPF in both the Better@Home cohort and historical control group. Healthcare costs during home-based GR consist of consultations with care professionals as well as additional healthcare costs. Consultations with care professionals (i.e., physical therapists, occupational therapists, dietitians, speech therapists, physicians, psychologists, nurses, and others) during home-based GR are registered on registration forms by the care professionals and include treatment volume, treatment time, and travel time. Furthermore, the nature of these consultations (at home, outpatient, or remotely using eHealth) is also registered by the care professionals. Additional healthcare costs during home-based GR are collected in a face-to-face interview with the patient after completion of the GR trajectory and include home care, domestic care, hospital care (both inpatient and outpatient), and general practitioner consultations. No data on healthcare costs during home-based GR was gathered for the historical control group, as this was an exclusion criterion.

Regarding patient and family costs during inpatient GR, the costs of informal care are assessed by multiplying an estimated volume of informal care provided during inpatient GR with a standardized reference price per hour [36, 37] in both cohorts. In addition, patient and family costs during home-based GR consist of the volume of informal care received by the patient, which is assessed during the face-to-face interview with the patient post-GR. Travel expenses are calculated by multiplying the number of visits to a healthcare service with standardized distances and transportation prices, including parking fees. Both standard distances and transportation prices are provided by the manual for cost research and reference prices [36, 37].

#### *Follow-up evaluation* 

A cost analysis is performed in the *follow-up* evaluation which assesses the course of costs in the Better@Home cohort from GR admission up to three months post-GR. In addition to the costs assessed during inpatient and home-based GR, the healthcare-, family & patient costs made during the three months of follow-up are assessed by a face-to-face interview with the patient. These costs include home care, domestic care, hospital care (inpatient and outpatient), general practitioner consultations, mental healthcare utilization, utilization of allied care professionals, use of assistive devices, and informal care utilization.

#### Both studies

 For both the *comparative* and *follow-up* evaluation, costs are calculated by multiplying the volume with the reference price of the unit obtained from the Dutch manual for economic evaluations from 2024 [36, 37]. Discounting is not applied in this study because to the total follow-up time (i.e., from GR admission to completion of GR trajectory) is expected to be shorter than 6 months.

#### Mixed-methods feasibility study

The feasibility study is conducted with a mixed method approach. It assesses the feasibility of the Better@Home program, as perceived by patients in the Better@Home cohort, their primary informal caregivers, and care professionals involved in their GR treatment. Feasibility is operationalized according to the method of Saunders [38], focusing on the following aspects: performance of the program according to plan, including the extent to which all core elements of the program were provided to the target population (fidelity and dose delivered completeness); extent to which the intended target group for the pathway was reached (reach); the extent to which the patients and informal caregivers actively engaged in the program (dose received exposure); barriers and facilitators that may have influenced the implementation of the program (context); and overall opinion of patients, informal caregivers and care professionals with the program (dose received satisfaction).

Data is collected among the patients of the Better@Home cohort using face-to-face structured interviews and among the informal caregivers using self-administered questionnaires. These structured interviews and questionnaires are administered at admission to GR, after completion of the GR trajectory, and after three months of follow-up. Data among the care professionals are gathered using registration forms, which the care professionals administer after providing home-based therapy and during or after weekly multidisciplinary team meetings. In addition, semi-structured group interviews (one group interview per GR facility) are conducted about two months after the inclusion of new patients in the study is closed. The group interviews are conducted with multidisciplinary project group representatives who led the Better@Home implementation in the eight participating GR facilities. Table 3 presents the data collected for the mixed-methods feasibility study.

**Table 3:** Data collection mixed-methods feasibility study Better@Home cohort

Process domain/indicators	Operalization	Admission GR	Post GR	Follow-up 3 months	2 months after closing inclusion
**Reach** Extent to which the intended target group for the pathway was reached	The number who refused, dropped out or completed the pathway.	RF	RR	RR	
	Reasons for refusals and dropouts	RF	RR	RR	GI
**Fidelity and dose delivered completeness** Implementation according to plan	Replace part of inpatient GR with home-based GR	FI	RF FI		GI
	Focus on participation goals inpatient and home-based	FI	RF FI		GI
	Use eHealth to support rehabilitation		RF FI		GI
	Promote self-management		RF		GI
	Close collaboration between all care partners	SQ	RF		GI
**Dose received exposure** Extend to which the patient and informal caregivers actively engaged in the programme	Replace part of inpatient GR with home-based GR		RF FI SQ		
	Focus on participation goals inpatient and home-based	FI	FI SQ		
	Use eHealth to support rehabilitation	FI	FI SQ	FI	
	Self-management		FI SQ	FI SQ	
**Dose received satisfaction** What is the opinion of rehabilitants, their relatives, and healthcare professionals about the programme	Replace part of inpatient GR with home-based GR	SQ FI	RF FI		GI
	Focus on participation goals inpatient and home-based		RF FI		GI
	Use eHealth to support rehabilitation		RF FI		GI
	Promote self-management		RF FI		GI
	Close collaboration between all care partners		FI SQ	FI SQ	GI
**Context** What were barriers and facilitators influencing implementation of the programme	Replace part of inpatient GR with home-based GR				GI
	Focus on participation goals inpatient and home-based				GI
	Use eHealth to support rehabilitation		RF		GI
	Promote self-management		RF		GI
	Close collaboration between all care partners				GI

*PF* Patient file, *FI* Face to face interview patient, *RF * registration form care professionals, *RR* Registration Researchers, *SQ *structured questionnaire primary informal caregiver patient, *GI* Group interview care professionals, *GR* Geriatric Rehabilitation

#### Background characteristics

The following background characteristics are collected among the patients in both the Better@Home cohort and historical control group, at admission to the GR facility: age, sex, living arrangement (alone or with others), and diagnosis relevant for rehabilitation (see Table 2 ). Furthermore, in the Better@Home cohort, the following additional background characteristics are collected during the face-to-face interview: educational level and frailty. Frailty is assessed by the Groningen Frailty Indicator (GFI) [39]. The GFI is a 15-item screening instrument to determine the level of frailty. It measures the loss of functions and resources in four domains: physical (mobility functions, multiple health problems, physical fatigue, vision, and hearing), cognitive (cognitive dysfunction), social (emotional isolation), and psychological (depressed mood and feelings of anxiety). All answer categories are dichotomized, and a score of 1 indicates a dependency problem. The range of the GFI score is 0–15. A score of 4 or higher represents moderate-to-severe frailty [40].

In addition, among the informal caregivers of the patients in the Better@Home cohort, the following background characteristics are measured at the patient’s admission using a self-administered questionnaire: age, sex, relationship to the patient, and travel distance to the patient.

Lastly, among the care professionals who filled in the registration forms, we collect discipline, and among the care professionals participating in the group interview we collect age, sex, discipline, and role in the Better@Home program.

### Data analysis

#### Outcome assessment

Statistical analyses will be performed using the statistical software package IBM SPSS Statistics. For both the *comparative evaluation* and the *follow-up evaluation*, descriptive statistics will be used to describe the background characteristics and scores on the outcome measures at admission to the GR facility of patients and informal caregivers (only in the Better@Home cohort). Furthermore, model assumptions (e.g. normality, linearity) will be checked for each model. If a model assumption is violated, appropriate remedies are considered. Missing data on the outcome variables will be addressed by using mixed models, with missing data on the covariates being addressed by using multiple imputation if the percentage of missing values is more than 5%. Both mixed models and multiple imputation yield unbiased and efficient estimates under the missing at random (MAR) assumption. Lastly, the analysis models for both the *comparative* and *follow-up* evaluations adjust for GR facility using dummy coding, which corresponds to a fixed-effects approach. This method fully accounts for between-center variability [41]. Within-center variability is addressed by adjusting for individual-level characteristics in the models, including age, sex, living situation, and diagnosis. Additionally, frequency matching between the Better@Home cohort and historical control group helps to further the balance. However, while confounding at the center level is not an issue thanks to a fixed-effect approach, we cannot be certain that some relevant confounder is omitted at the subject-level.

#### Comparative evaluation

 A linear two-level mixed model (LMM) will be used, which includes independence in daily functioning (BI) as a dependent variable and GR group (Better@Home versus Care as usual), time, time by GR group interaction and facility by GR group interaction as independent variables. Furthermore, facility, age, sex, living situation (alone versus with others), and diagnosis will be covariates. The choice for a LMM is due to the hierarchical nature of the data and the repeated measures design. Each level in the hierarchy is a contextual variable, and each contextual variable has dependency in the data, which means that residuals will be correlated. For this study, the repeated measures (in time) of the BI is a level 1 variable; the subjects (patients) and their characteristics are the level 2 variable, and the facilities are the level 3 variable. In our LMM analysis, the time factor will be used as a continuous variable (due to the differences between patients in their duration of the GR trajectory). Given that the number of independent variables is limited and that the research question focuses on specific variables (i.e., group in the comparative evaluation, time in the follow-up study), the LMM will be built top-down, starting from the most complex model.

#### Follow-up evaluation

 Similar to the comparative evaluation, a LMM will be used. Here, the COPM-p will be the dependant variable with the COPM-s, BI, and EQ-5D-5 L as secondary outcomes. GR group (Better@Home versus Care as usual), time, time by GR group interaction and facility by GR group interaction are the independent variables. Furthermore, facility, age, sex, living situation (alone versus with others), and diagnosis will be the covariates.

#### Cost assessment

*Comparative evaluation.* For the cost-effectiveness study, descriptive statistics are used to present mean volumes and costs of healthcare use at baseline. Baseline costs are compared with non-parametric bootstrapping (5,000 times). Statistically significant differences in costs are determined using a 95% Confidence Interval. If the value ‘0’ is included in the confidence interval, this indicates no cost difference between the groups. The incremental cost-effectiveness ratio (ICER) expressed in cost per unit of activities of daily living is estimated as the difference in outcomes on the BI between the Better@Home cohort and historical control group divided by their differences in costs. To characterize sampling uncertainty when estimating the ICER, the costs and effects are also bootstrapped (5,000 times). The resulting 5,000 cost-effectiveness ratios are presented on two incremental-cost effectiveness planes with four quadrants. When performing bootstrap analyses, a higher score represents a positive outcome. Therefore, only for bootstrapping purposes, the BI scores are reversed (a higher score representing less dependence on activities of daily living). Additional sensitivity analyses are conducted to evaluate the robustness of the results. These analyses will at least include a complete case analysis and an analysis of the healthcare perspective instead of the societal perspective. Lastly, given the observational design, the analysis aims to explore whether home-based GR is associated with comparable or improved outcomes at lower or similar costs. If home-based GR results in cost savings with comparable outcomes, a cost-minimization approach will be applied. If both incremental costs and outcomes are positive, the incremental cost-effectiveness ratio (ICER) will be estimated to assess the additional cost per unit of effect.

*Follow-up study.* The course of the costs in the two measurement periods (i.e. the period from GR admission to post-GR, after completion of the GR trajectory; and three months of follow-up post-GR) are analyzed using descriptive statistics.

#### Mixed-methods feasibility study

Descriptive statistics are used to analyse quantitative data from the feasibility study in the Better@Home cohort, and thematic analysis is used to analyse qualitative data [42].

### Progress of the study

The Better@Home program was implemented in the eight participating GR facilities between November 2023 and March 2024. Participant inclusion started in January 2024 (for those facilities that already had implemented the Better@home program) and continued until November 2024. Data collection continues until April 2025, and results are expected to be available for publication at the end of 2025.

## Discussion

Implementing home-based GR is crucial for making GR sustainable in the future [8, 9]. However, implementation faces many challenges, including regulatory and reimbursement issues, as well as a lack of knowledge and skills. There is still insufficient practical knowledge about the effective execution of home-based GR, highlighting the need for further development and research.

This paper presents a study protocol aiming to assess the (cost-)effectiveness and feasibility of home-based GR after (shorter) inpatient GR.

This study involves collaboration between eight GR facilities in three regions in the Netherlands that implement and evaluate the Better@Home program. The study results will be valuable to healthcare organizations providing GR, policymakers, health insurers, patient representatives, and other stakeholders by supporting informed decisions on implementing future-proof home-based GR and identifying potential areas for further program optimization. Furthermore, the study will offer valuable insights into new opportunities for patients undergoing GR. Many patients only fully recognize the impact of their limitations and impairments after being discharged from inpatient GR [7]. The transition from inpatient care to the home environment is often challenging; however, home-based GR may ease this process by enabling patients to engage in meaningful daily activities within their own homes, supported by a multidisciplinary team of healthcare professionals. This approach has the potential to help patients and their informal caregivers develop sustainable strategies for daily participation, ultimately extending the period of independent functioning despite ongoing impairments. Because of the multicentre design, this study allows for the comprehensive evaluation of outcomes, costs, and feasibility of home-based GR. It is expected that useful information on the feasibility of the program, such as the program’s reach, adherence, and fidelity, and insight into barriers and facilitators encountered when providing home-based GR, will be provided. These insights will contribute to further shaping, optimizing, and implementing a future-proof home-based GR.

A number of limitations of the design of the *prospective cohort study* are present. First, only one outcome measure and a limited number of background characteristics are available in the historical control group, presenting limitations in the ability to control for potential confounding variables and to fully assess the comparability between groups in the comparative evaluation. This may compromise the internal validity of the study’s findings. Second, there is a reasonable risk of selection bias, although measures are taken to reduce this risk and its consequences by the frequency matching procedure. Third, a randomized or experimental design was considered but deemed not feasible for this study. The study aligns with current developments in healthcare, where home-based geriatric rehabilitation is already being implemented, albeit on a limited scale, as part of usual care. Withholding home-based geriatric rehabilitation from patients who would otherwise be eligible for this care was considered unethical, making randomization inappropriate in this context. As a result, we opted for a historical control group design, recognizing that this decision involves methodological trade-offs, including the potential for residual confounding.

A limitation of the *prospective cohort study* is the lack of historical control group data beyond the completion of GR. While it is still possible to provide insight into the course of outcomes and costs within the Better@Home cohort from GR admission to three months after the completion of GR, the *follow-up evaluation* does not offer the possibility of drawing conclusions regarding the long-term outcomes and costs of the Better@Home program. Moreover, it is important to note that, given the observational design of this study, associations observed within the study context do not imply definitive causal relationships. While measures have been taken to minimize bias, including matching procedures and adjusted analyses, the non-randomized nature of the comparative evaluation requires caution in interpreting our findings, particularly in terms of causality.

If this study shows promising results on the feasibility, outcomes, and costs of the Better@Home program, the aim is to upscale the intervention into Dutch healthcare. When the results are less promising, the study might provide helpful input for further optimization of home-based GR. Although some methodological limitations concerning the current study exist, the presented study protocol is currently considered the most feasible method to gain insight into the (cost-)effectiveness and feasibility of the Better@Home program among this population of multimorbid and frail older persons in need of GR. The presented study protocol may provide a solid base for further optimizing, fine-tuning and (re)evaluating the Better@Home program in the future.

### Status of data collection

Participant recruitment started in February 2024 and will run through April 30, 2025. At the time of submission (November 2024), 109 participants had already been enrolled in the study.

## Supplementary Information

Supplementary Material 1.

Supplementary Material 2.

## Data Availability

Anonymized datasets generated and analyzed as part of this study may be available by the corresponding author upon reasonable request after completion of the study.

## Abbreviations

GR: Geriatric Rehabilitation
Home-based GR: Home-based geriatric rehabilitation
Better@Home: name of a Home-based geriatric rehabilitation program
AWO-L: Limburg Living lab in Ageing and Long-term Care
UNO Amsterdam: University Network of Organizations for Care for Older Adults
UNC-ZH: University Network for the Care Sector South-Holland
COPM: Canadian Occupational Performance Measure
BH: Better@Home cohort
CU: Historical Care as usual cohort
PF: Patient file
FI: Face face-to-face interview patient
RF: Registration form care professionals
RR: Registration Researchers
SQ: Structured questionnaire primary informal caregiver
GI: Group interview care professionals
BI: Barthel-index
COPM-p: Canadian Occupational Performance Measure-performance measure
COPM-s: Canadian Occupational Performance Measure-satisfaction measure
EQ5D: EuroQol health-related quality of life
GFI: Groningen Frailty scale
CSI: Caregiver Strain Index
USER: Utrecht Scale for Evaluation of Rehabilitation
ICER: Incremental Cost-Effectiveness Ratio
